# Long Non-coding RNA DLEU2L Targets miR-210-3p to Suppress Gemcitabine Resistance in Pancreatic Cancer Cells via BRCA2 Regulation

**DOI:** 10.3389/fmolb.2021.645365

**Published:** 2021-04-22

**Authors:** Fei Xu, Heshui Wu, Jiongxin Xiong, Tao Peng

**Affiliations:** ^1^Department of Gastrointestinal Surgery, Union Hospital, Tongji Medical College, Huazhong University of Science and Technology, Wuhan, China; ^2^Department of Pancreatic Surgery, Union Hospital, Tongji Medical College, Huazhong University of Science and Technology, Wuhan, China

**Keywords:** lncRNA, competing endogenous RNA, drug resistance, microRNA, Warburg effect, cancer metabolism

## Abstract

Gemcitabine (GEM) resistance remains a challenging clinical issue to overcome in chemotherapy against pancreatic cancer. We previously demonstrated that miR-210 derived from pancreatic cancer stem cells enhanced the GEM-resistant properties of pancreatic cancer cells, thus identifying miR-210 as an oncogenic miRNA. Herein, we report the existence of an upstream effector that acts as a competing endogenous RNA (ceRNA) to miR-210. Bioinformatic screening was performed to identify lncRNAs with a binding relationship to miR-210. Overexpression and interference vectors were constructed to demonstrate the effect of ceRNA activity in pancreatic cell behavior, both *in vitro* and *in vivo*. DLEU2L (deleted in lymphocytic leukemia 2-like), which is expressed at low levels in pancreatic cancer tissues, was shown to exhibit a binding relationship with miR-210-3p. Overexpression of DLEU2L and silencing of miR-210-3p suppressed the proliferation, migration, and invasion of pancreatic cancer cells while promoting apoptosis. These effects occurred via the inhibition of the Warburg effect (aerobic glycolysis) and AKT/mTOR signaling. In addition, we showed that BRCA2 is a target gene of miR-210-3p, and the downregulation of miR-210-3p by DLEU2L effectively induced an upregulation of BRCA2 via the ceRNA mechanism. *In vivo*, DLEU2L overexpression and miR-210-3p interference suppressed pancreatic tumor progression, consistent with the results of *in vitro* studies. The findings of our study establish DLEU2L as a ceRNA to miR-210-3p and reveal the critical role of the DLEU2L/miR-210-3p crosstalk in targeting GEM resistance.

## Introduction

Pancreatic cancer is a deadly malignancy with rising prevalence, poor prognosis, and low overall survival rates due to distant metastasis at diagnosis (Siegel et al., 2019). In 2019, there were an estimated > 55,000 new cases of pancreatic cancer and > 45,000 cases of related death in the United States alone (Siegel et al., 2019). Gemcitabine (GEM, 2′,2′-difluorodeoxycytidine) is a nucleoside analog that exerts anti-cancer action by incorporating into genomic DNA to inhibit the process of DNA synthesis (Bouffard et al., 1993; Plunkett et al., 1995). It has been clinically applied as a first-line chemotherapeutic drug for pancreatic cancer, but drug resistance, both intrinsic and acquired, remains a challenge that obstructs the effective use of this drug (Amrutkar and Gladhaug, 2017). There is thus a need to develop strategies to overcome chemoresistance and render tumor cells sensitive to chemotherapeutic treatment.

Recent years have seen the emergence of strategies in targeted therapy that aim to induce effective cancer treatment by recognizing specific tumor biomarkers. For example, the suppression of identified oncogenes and the activation of tumor-suppressors are promising ways of impairing tumor growth and development, thus slowing disease progression (Hong et al., 2014; Waters and Der, 2018). Tumor biomarkers include a variety of biomolecules such as proteins, DNA, and RNA. Among these, microRNAs (miRNAs) and long non-coding RNAs (lncRNAs) have gained widespread attention as tumor biomarkers (Chen et al., 2015; Arun et al., 2018). miRNAs are generally 18–25 nucleotides in length while lncRNAs are usually > 200 nucleotides in length. Despite this difference, both miRNAs and lncRNAs have the ability to target mRNA in tumor cells to suppress their expression, thereby exhibiting either oncogenic or tumor-suppressing potential depending on the properties of the target gene. In addition, lncRNAs can act as competing endogenous RNAs (ceRNAs) by binding to miRNAs, in turn regulating the target genes of the miRNAs via a “sponging” effect. For example, the lncRNA SNHG16 was shown to promote pancreatic cancer growth by sponging miR-218-5p (Liu et al., 2019). Similarly, the lncRNA GSTM3TV2 plays an oncogenic role in pancreatic cancer by sponging let-7, thereby upregulating L-type amino acid transporter 2 and oxidized low-density lipoprotein receptor 1 to promote GEM resistance (Xiong et al., 2019).

We have previously demonstrated the function of miR-210 as an oncogenic miRNA that mediates chemoresistance to GEM via the protein kinase B/mammalian target of rapamycin (AKT/mTOR) signaling pathway in multiple pancreatic cancer cell lines and in an *in vivo* animal model (Yang et al., 2019). miR-210 has been shown to play critical roles in tumor progression by affecting cellular phenomena such as epithelial-mesenchymal transition (Ni et al., 2019), autophagy (Liu et al., 2020), and angiogenesis (Yang et al., 2016). However, it is unknown whether miR-210 interacts with a specific target gene to promote drug resistance in pancreatic cancer. Further, whether miR-210 can be regulated by lncRNAs through sponging mechanisms remains to be elucidated. Herein, we identified DLEU2L (deleted in lymphocytic leukemia 2-like) as a tumor-suppressing lncRNA that exhibits a binding relationship with miR-210, thereby downregulating miR-210 upon interaction. We evaluated whether the overexpression of DLEU2L impairs pancreatic cancer progression *in vitro* and tumor development *in vivo* using a nude mouse xenograft model, by examining cancer cell proliferation, apoptosis, glucose metabolism, drug resistance, and molecular signaling pathways.

## Materials and Methods

### Bioinformatics Pre-screening

The RNA22 tool^1^ was employed to screen for lncRNAs that have a potential binding relationship with miR-210-3p. Data on the expression of DLEU2L in pancreatic tumor tissues and normal adjacent tissues were obtained from the GEPIA web site^2^ using clinical samples in The Cancer Genome Atlas database.

### Cell Culture, Transfection, and GEM Treatment

The human pancreatic cell line PANC-1 was acquired from the cell bank of the Chinese Academy of Sciences (Shanghai, China) and cultured in Dulbecco’s modified Eagle medium (DMEM, SH30022.01B, Hyclone, Carlsbad, CA) containing 10% fetal bovine serum (FBS, 10270-106, Gibco, Waltham, MA) in an atmosphere containing 5% CO_2_ at 37°C. For transfection, DLEU2L overexpression and miR-210-3p interference vectors were prepared using sequences synthesized by Wuhan Tianyi Huiyuan Bioscience and Technology Inc. (Wuhan, China). The overexpression and interference sequences are shown in Table 1. PANC-1 cells were transfected with either DLEU2L overexpression (ov-DLEU2L) or miR-210-3p interference (sh-miR-210-3p) fragments or the corresponding negative controls (ov-NC or sh-NC). After transfection, PANC-1 cells were treated with GEM (S1714, Selleck Chemicals, Houston, TX) dissolved in dimethyl sulfoxide at 0, 0.5, 1.5, or 15 μM for 48 h for western blot; 24, 48, or 72 for 3-(4,5-dimethylthiazol-2-yl)-2,5-diphenyltetrazolium bromide (MTT) assay; or 24 h for flow cytometry.

**TABLE 1:** Overexpression and interference sequences for transfection.

	Sequence
**DLEU2L overexpression**	ACCTGATCTCATCAATCTAGCGGAAGAGACAGGATAACC TATCCA AGAGTATAGCGCCACTATGACTCCGCCGGAAAAATTACTT TAAAA ATCGCCAAAAATTACTTGGAGCAAAGGGCAGTAGGCGG AGCTTCG CCAAGGCTGGCGCAGTCGGTTTTGACCTGTAGCAGAGA ACCAATT CTGGAGAACAGCCTCACTTCTTTGATTGAATACTCACATA ATGCT TTGGAACAGGACATGAGATTAAGGTTTAATAATGATAGAAT GAAG ACCATAATAAAAGAGACCTCTACTTACCTCAGCAATTCTTA CCTT TCTTACCTATTTGATGAAGATGTCTTTTGAAAGGTGTACTG CAAG GAACAAAATGTTTGTAAATTCTGCTTTTACCAAGGTTTCTT TTTT AGTTGATGCCAAAGAGTTCCAATATTGAACATCTTAAGTC TGTTA CTTGGAGTATGGATTGAGTTTGGAGCTTACTCAGAGGAC TACAGG AGAGTATCCAGGAGGTGGATAATTACTGTACCTTCCTCAT GGAAA AAGTTTTATTTAAAGTGTTATTTCTCGTTGAATACTATCAAA AAG GAAAAAAAAATGACCTAAACTTTTGAGACAGATTTGGCTC TAGTA AGTATTTAGATATATCGCTTGCATATCTGGGAGAAGAAATA AGAG ACTATCATCAATACATTCCCATCTACTAAAAAATTTTATTTT ACA CATGTCAAGGGATTACTTATAACTTCCATTGTCTTTTTTTTA AGC CTATATGCAATGAAAAATATATTGGCAAAATAAATATTTAAA CCT TTTATGTTAAAATTACTGTGAAAGATGACAAGTTAGCTGCT GTTT TTGTCTACATTATACTGAAATTAAATGTTTATAATTTATATT TTG GGTTTATTTATTTATAAATCATGGAATTTATGCAAAAAACAT GAG TAGTACAGATTCTCCTCTAATTCTGTAGGACTTTGAATAAT GTGA TATTTTTCTTATAGTTGGACCCTTGTGTTTTGAAGAAATGC CAAC TGCTTGAAGAATTTCCTTGTTATTTGTATTATTTGCTATAGG GTT AGATGTTGAGAAATTCTTCTGACAAAAAATTTTAAGCCAG TTTTA CACTAAATATTCCTTAGTCTGATTAATTTGTTATTGGATGTA TTC TGTATCTTTCTTTTGTAATTTGTAACTTTTATCCACTTAGCA CGA ATGATTCTATTGAAGAAAATCTTTAGGAAGTGGTAGAAACT TTAA ATCGCCCCATAGTTTGCCTGTTTCCACATTTTATTATCTTAT AAT CTTTGGGAGTGCTTACACTTATGGAGCTAACATTTTCAGA GATAC AGGTTCTCATAGTACTACTAAAACTTTTTTCCTCTTTGGAC TGAA TACCTATGATTATAACTATACAGTAGTTTAAGTTTCCTTGT GATT
	AGTCAAAAATACCATTTTAGTATGAAGCAATGAAGTCTATT ATTT GTTGTCCCATAATTGAGAAACTTAGATACACCTTTTATTAA GAGT TTGTAAATTCTAGCTTAGTCTACACAGATTTTTATATCAATT TGT TTATATTTTTATTAATGTCATTTCTGGAATTGTGAAAATGTT AAT GTTCGACAAGCAACATTAAAAATAGATTTGAAACATT
**miR-210-3p interference**	TATTGCACTTGTCCCGGCCTGT

### Dual Luciferase Reporter Assay

RNA22 (see text footnote 1) and starBase V2.0^3^ were employed to screen for potential target lncRNAs and genes of miR-210-3p, among which DLEU2L and breast cancer type 2 susceptibility protein (BRCA2) were selected, respectively. Binding sites between miR-210-3p and DLEU2L or the 3′ untranslated region (UTR) of BRCA2, were identified, and wild-type (WT) and mutant (MUT) 3′UTR segments of the binding sites were prepared based on the predicted sequences. Transfection was performed with pmirGLO plasmids (Addgene, Watertown, MA) using the LucPair^TM^ Duo-Luciferase Assay Kit (LF001, GeneCopoeia, Rockville, MD). After transfected cells were cultured with miR-210-3p mimics or negative control (NC), the signal of firefly luciferase and renilla luciferase was detected using a Glo-MAX 20/20 analyzer.

### Transwell Assay

To evaluate the migration and invasion capability of PANC-1 cells after transfection, Transwell assays were carried out. Transwell chambers (Corning Inc., Corning, NY) and 24-well plates were first soaked with 1 × phosphate-buffered saline (PBS) for 5 min. For invasion assays, the top Transwell chambers were pre-coated with 80 μL of Matrigel (354230, BD Biosciences, Franklin Lakes, NJ) for 30 min at 37°C (chambers were non-coated for migration assay). Before the experiment, transfected cells were incubated in serum-free DMEM for 24 h. The cells were then trypsinized and suspended at 1 × 10^5^ cells/mL in DMEM containing 1% FBS. In the lower Transwell chambers, 750 μL of DMEM containing 10% FBS was added in each well of a 24-well plate, and 500 μL of cells were seeded on the top chamber. The plate was incubated at 37°C for 24 h, and 1 mL of 4% formaldehyde solution was added to each well for 10 min of fixation. After the solution was removed, the cells were washed once with 1 × PBS and 1 mL of 0.5% crystal violet solution (C1701, Bioswamp, Wuhan, China) was added to each well. The cells were stained for 30 min and washed three times with 1 × PBS. After drying, the non-migrated cells were removed using a cotton swab and the cells were visualized using an optical microscope for cell counting.

### Western Blot

Total proteins were extracted from cell or tissue samples using radioimmunoprecipitation assay buffer containing protease and phosphatase inhibitors and quantified using a bicinchoninic acid assay. Sodium dodecyl sulfate-polyacrylamide gel electrophoresis was performed using 20 μg of denatured proteins in each lane, after which the proteins were transferred to polyvinylidene fluoride membranes. Blocking was performed by incubating the membranes in 5% skimmed milk for 2 h at room temperature. After blocking, the membranes were immersed in diluted primary antibodies (Table 2) overnight at 4°C and washed three times with PBS/Tween 20 (PBST) for 5 min each. Then, the membranes were incubated in horseradish peroxidase-conjugated secondary antibodies for 1 h at room temperature and rinse three times with PBST for 5 min each. An enhanced chemiluminescence reagent (WBKLS0010, Millipore, Burlington, MA) was added for color detection using a Tanon-5200 automatic analyzer (Tanon, Shanghai, China), and the protein bands were visualized and analyzed using Tanon GIS software.

**TABLE 2:** Antibodies for western blot.

Protein	Full name	Supplier	Product code	Dilution
GLUT1	Glucose transporter 1	Bioswamp	PAB30639	1:1,000
LDHB	Lactate dehydrogenase B	Bioswamp	PAB30703	1:1,000
HK2	Hexokinase 2	Bioswamp	PAB30271	1:1,000
PKM2	Pyruvate kinase isozyme M2	Bioswamp	PAB42174	1:1,000
Bax	Bcl2-associated X protein	Bioswamp	PAB30040	1:2,000
Bcl-2	B-cell lymphoma 2	Bioswamp	PAB30041	1:2,000
Cyt-c	Cytochrome-c	Abcam	ab90529	1:1,000
Casp-3	Caspase 3	Bioswamp	PAB30665	1:2,000
AKT	Protein kinase B	Bioswamp	PAB30596	1:1,000
p-AKT	Phosphorylated protein kinase B	Bioswamp	PAB43116-P	1:1,000
mTOR	Mammalian target of rapamycin	Bioswamp	PAB30674	1:1,000
p-mTOR	Phosphorylated mammalian target of rapamycin	Bioswamp	PAB36313-P	1:1,000
p-4EBP1	Phosphorylated eukaryotic initiation factor 4E-binding protein	Bioswamp	PAB36285-P	1:1,000
4EBP1	Eukaryotic initiation factor 4E-binding protein	Bioswamp	PAB31069	1:1,000
S6K	Ribosomal protein S6 kinase	Bioswamp	PAB33261	1:1,000
p-S6K	Phosphorylated ribosomal protein S6 kinase	Bioswamp	PAB43272-P	1:1,000
BRCA2	Breast cancer gene 2	Bioswamp	PAB30554	1:1,000
GAPDH	Glyceraldehyde 3-phosphate dehydrogenase	Bioswamp	PAB36269	1:1,000
Goat anti-rabbit IgG	N/A	Bioswamp	SAB43714	1:20,000

### Biochemical Assays

Adenosine triphosphate (ATP) content (product code: A095) and lactic acid production (product code: A019-2) were evaluated using respective assay kits provided by the Nanjing Jiancheng Bioengineering Institute (Nanjing, China). Glucose uptake was assessed using an assay kit (product code: KA4086) provided by Abnova (Taipei, Taiwan). All required reagents were provided in the kits and the assays were performed according to the manufacturer’s instructions.

### MTT Assay

Transfected PANC-1 cells were seeded in a 96-well plate at 5 × 10^3^ cells/well (180 μL/well). The plate was incubated overnight at 37°C in an atmosphere containing 5% CO_2_ for the cells to adhere. The cells were then treated with GEM (0, 0.5, 1.5, or 15 μM) for 0, 24, 48, or 72 h. After GEM treatment, 20 μL of 5 mg/mL MTT solution (PAB180013, Bioswamp) was added to each well and the cells were further incubated for 4 h. Thereafter, the solution in the wells was aspirated and 150 μL of dimethyl sulfoxide was added to each well. The plate was gently shaken for 10 min and the absorbance of the wells was measured at 490 nm using a plate reader to evaluate relative cell proliferation.

### Flow Cytometry

Transfected and/or GEM-treated (24 h) PANC-1 cells were washed once with PBS and trypsinized. The cells were centrifuged at 1,000 × g for 5 min, the supernatant was removed, and the cells were resuspended in PBS. To assess apoptosis, 1 × 10^6^ cells were centrifuged at 1,000 × g at 4°C for 5 min. The supernatant was removed and 1 mL of cold PBS was added to resuspend the cells gently. This step was performed three times in total. After the final centrifugation, the cells were resuspended in 200 μL of binding buffer, along with 10 μL of annexin V-fluorescein isothiocyanate solution and 10 μL of propidium iodide, both provided in an apoptosis assay kit (556547, BD Biosciences). The mixture was gently mixed and incubated at 4°C in the dark for 30 min, and 300 μL of binding buffer was added. Flow cytometry was carried out and the data were analyzed using NovoExpress software (NovoCyte, ACEA Biosciences, Inc., San Diego, CA). To examine cell cycle progression, the suspended cells were fixed in 700 μL of anhydrous ethanol at −20°C for 24 h. The fixed sample was centrifuged at 3,000 × g for 30 s and the supernatant was removed. Then the precipitated cells washed twice with 1 mL of cold PBS. The precipitate was resuspended in 100 μL of 1 mg/mL RNAse A and the cells were incubated at 37°C to allow RNA to degrade, and 400 μL of 50 μg/mL propidium iodide was added. After 10 min of incubation in the absence of light, DNA content was determined using flow cytometry and the proportion of cells at each phase of the cell cycle was evaluated using NovoExpress software.

### Animal Model of Xenografted Pancreatic Tumor

All animal experiments were performed in accordance with the Guidelines for Animal Care and Use of the Model Animal Research Institute and was approved by the ethics committee of Wuhan Myhalic Biotechnology Co., Ltd. (approval number HLK-20190225-01). The *in vivo* model of xenografted pancreatic cancer was established in thirty male BALB/c nude mice aged 4 weeks and weighing 18–20 g (obtained from Cavens Laboratories, Changzhou, China). The mice were housed in specific-pathogen-free conditions (22–26°C, 50–60% relative humidity) in a 12/12-h light/dark cycle and given free access to food and water. After a 7-day initial adaptive period, the mice were randomly divided into five groups (*n* = 6 per group): Control (non-transfected), ov-DLEU2L (DLEU2L overexpression), ov-NC (empty overexpression vector as negative control), sh-miR-210-3p (miR-210-3p interference), and sh-NC (empty interference vector as negative control). Based on the respective groups, the mice were subjected to subcutaneous injection of non-transfected or transfected (DLEU2L overexpression or miR-210-3p interference vectors, or their respective negative controls) PANC-1 cells at 1 × 10^6^ cells/mL at the right armpit. After 21 days of tumor growth, the mice were sacrificed with an overdose of sodium pentobarbital and the tumors were removed for subsequent characterization.

### Quantitative Reverse Transcription Polymerase Chain Reaction (qRT-PCR)

RNA extraction was carried out using TRIzol reagent (15596026, Ambion, Inc., Foster City, CA). The extracted RNA was reverse-transcribed into cDNA using the RevertAid First Strand cDNA Synthesis Kit (K1622, Thermo Scientific, Waltham, MA) and TaqMan kit (Applied Biosystems, Foster City, CA). qRT-PCR was performed using the SYBR Green PCR kit (KM4101, KAPA Biosystems, Wilmington, MA). The primer sequences are listed in Table 3 and the experimental conditions are described in Table 4. Data were acquired using the QuantStudio^TM^ 6 Flex Real-Time PCR System (Applied Biosystems) and analyzed by the 2^–ΔΔCt^ method.

**TABLE 3:** Primers for qRT-PCR.

Primer	Sequence
DLEU2L forward	AAATTCTGCTTTTACCAAG
DLEU2L reverse	CTCCAAACTCAATCCATAC
miR-210-3p stem loop	CCACTCCTCCACCTTTG
miR-210-3p forward	CACCACCCTGTTGCTGT
miR-210-3p reverse	CTCAACTGGTGTCGTGGAGTCGGCAATTCAGTTGA GTCAGCCGC
GAPDH forward	GGGCTGTGCGTGTGACA
GAPDH reverse	AACTGGTGTCGTGGAGTCGGC
U6 forward	CTCGCTTCGGCAGCACA
U6 reverse	AACGCTTCACGAATTTGCGT

**TABLE 4:** Conditions for qRT-PCR.

Step	Temperature (°C)	Time (s)	Cycles
Initial denaturation	95	180	1
Denaturation	95	5	39
Annealing	56	10	
Extension	72	25	
Final extension	65	5	1
	95	50	

### Ki67 Staining

The extracted tumors were fixed in 10% neutral formalin for 2 days and embedded in paraffin wax. Before sectioning, the paraffin-embedded tissue block was hardened at −20°C for several minutes. The tissue block was cut into 2-μm-thick sections and fixed onto microscope slides. For immunohistochemical staining, the sections were heated for 1 h at 65°C and deparaffinized twice in xylene for 15 min each. The deparaffinized sections were soaked in a graded concentration series of ethanol (100% twice, 95, 85, and 75%) for 5 min at each concentration, then washed with running water for 10 min. Antigen retrieval was performed by immersing the sections in 0.01 M sodium citrate buffer for 15 min. After three washes with PBS for 3 min each, endogenous peroxidase removal was performed by immersing the sections in 3% H_2_O_2_ for 10 min. The sections were washed three times with PBS for 3 min each and blocked in 0.5 bovine serum albumin for 30 min. Then, the sections were incubated with Ki67 MaxVision^TM^ primary antibodies (PAB30684, Bioswamp) at 1:50 for 2 h at 37°C and washed three times with PBS for 3 min each. Thereafter, the sections were incubated with horseradish peroxidase-polymer anti-rabbit secondary antibodies (KIT-5020, MaxVision) at room temperature for 30 min and washed three times with PBS for 3 min each. Diaminobenzidine was added to the sections and when color change was observed, the sections were washed with running water to remove the dye. Hematoxylin counterstaining was performed for 3 min and differentiation was performed using 1% hydrochloric alcohol. The degree of staining was controlled under a microscope and the reaction was terminated by washing the sections under running water for 10 min. Then the sections were dehydrated in ethanol (75, 85, 95, and 100% twice) for 5 min at each concentration and transparentized by soaking twice in xylene for 3 min each. The sections were sealed with neutral balsam gum and analyzed using a microscope.

### Statistical Analysis

All numerical data are shown as the mean ± standard deviation (SD) of three replicates (*n* = 3) for *in vitro* experiments and six replicates (*n* = 6) for *in vivo* experiments. The one-sample *t*-test was performed to compare the means between two groups and one-way analysis of variance (ANOVA) followed by Tukey’s *post hoc* test was conducted to compare the means between more than two groups. *p* < 0.05 indicates statistical significance.

## Results

### DLEU2L Binds to miR-210-3p and Is Expressed at Low Levels in Pancreatic Cancer Tissues

**FIGURE 1:**
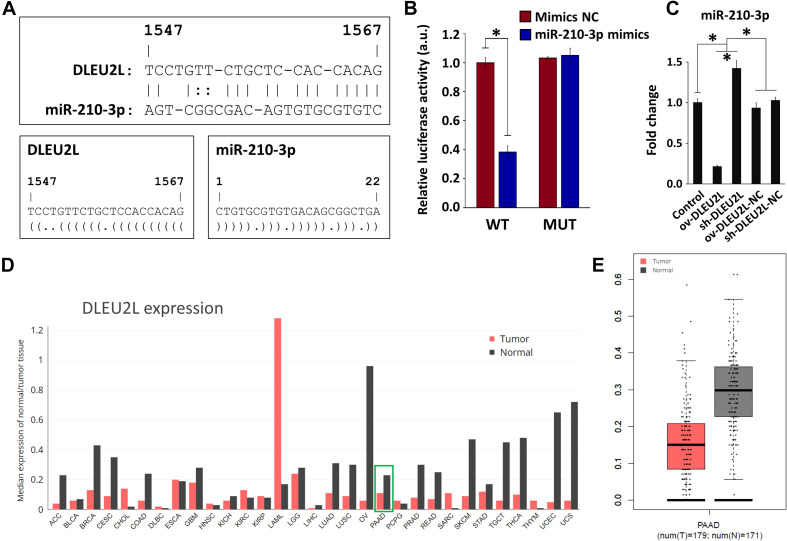
Targeting relationship between DLEU2L and miR-210-3p and expression of miR-210-3p in pancreatic cancer. **(A)** Binding sites between DLEU2L (from position 1,547) and miR-210-3p, predicted by RNA22. Dot-bracket notation illustrates binding at specific nucleotides. **(B)** Dual luciferase activity assay of binding relationship between DLEU2L and miR-210-3p, using miR-210-3p mimics or negative control (NC). Relative luciferase activity represents the ratio of firefly luciferase to renilla luciferase. **(C)** qRT-PCR of the expression of miR-210-3p in PANC-1 cells that were transfected with DLEU2L overexpression (ov-DLEU2L) or interference (sh-DLEU2L) vectors (or their respective negative control/NC). **(D)** Median expression of DLEU2L in various types of tumor tissues compared to that in adjacent normal tissues in clinical samples. The plot was generated using data from the GEPIA web site. **(E)** Relative expression of DLEU2L in PAAD tumor samples and adjacent normal tissue samples. The plot was generated using data from the GEPIA web site. PAAD, pancreatic adenocarcinoma. Numerical data in **(B,C)** are expressed as the mean ± SD (*n* = 3); ^∗^*p* < 0.05. Statistical analysis was carried out using ANOVA.

Using the RNA22 tool (Miranda et al., 2006), we screened for lncRNAs that exhibited binding sites with the oncogenic miR-210 (specifically miR-210-3p). This step was performed to identify potential miRNA-lncRNA targeting relationships. We revealed that miR-210-3p, which contains 22 nucleotides, binds to DLEU2L at position 1547 (Figure 1A). Through this prediction, we hypothesized that DLEU2L inhibits miR-210-3p expression by binding at the target site. To verify this hypothesis, we designed WT and MUT sequences of the DLEU2L binding site based on the prediction and performed a dual luciferase assay to determine whether binding occurs between miR-210-3p and DLEU2L. As anticipated, overexpression of miR-210-3p using mimics suppressed luciferase activity in WT-transfected cells (Figure 1B), successfully validating the binding between miR-210-3p and DLEU2L. We confirmed the relationship between DLEU2L and miR-210-3p by transfecting PANC-1 cells with DLEU2L overexpression (ov-DLEU2L) or interference (sh-DLEU2L) vectors (or their respective negative control/NC) and detecting the expression of miR-210-3p by qRT-PCR (Figure 1C). The results are consistent with those of luciferase activity assay, showing that ov-DLEU2L significantly downregulated the expression of miR-210-3p, whereas sh-DLEU2L significantly upregulated it. In other words, the targeting relationship was verified. Based on this result and our previous findings (Yang et al., 2019), we next assumed that DLEU2L plays a tumor-suppressing role, particularly in pancreatic cancer, as a target of (and in turn, by targeting) miR-210-3p. We used the GEPIA web server (Tang et al., 2017) to collect data on the median expression of DLEU2L in a variety of tumor types (Figures 1D,E). In clinical pancreatic adenocarcinoma (PAAD in Figure 1D) samples in The Cancer Genome Atlas database, DLEU2L was more highly expressed in adjacent normal tissues than in tumor tissues (the relative expression levels of DLEU2L and miR-210-3p in various tumor and normal tissues, detected in a pre-study, are shown in Supplementary Figure 1). Thus, we hypothesized that by overexpressing DLEU2L, miR-210-3p is conducive to over-binding with DLEU2L, thereby resulting in the suppression of its expression and the nullification or attenuation of its oncogenic function. The subsequent studies were designed to verify this hypothesis.

### DLEU2L Overexpression or miR-210-3p Interference Inhibited PANC-1 Cell Migration/Invasion and Altered Glucose Metabolism

**FIGURE 2:**
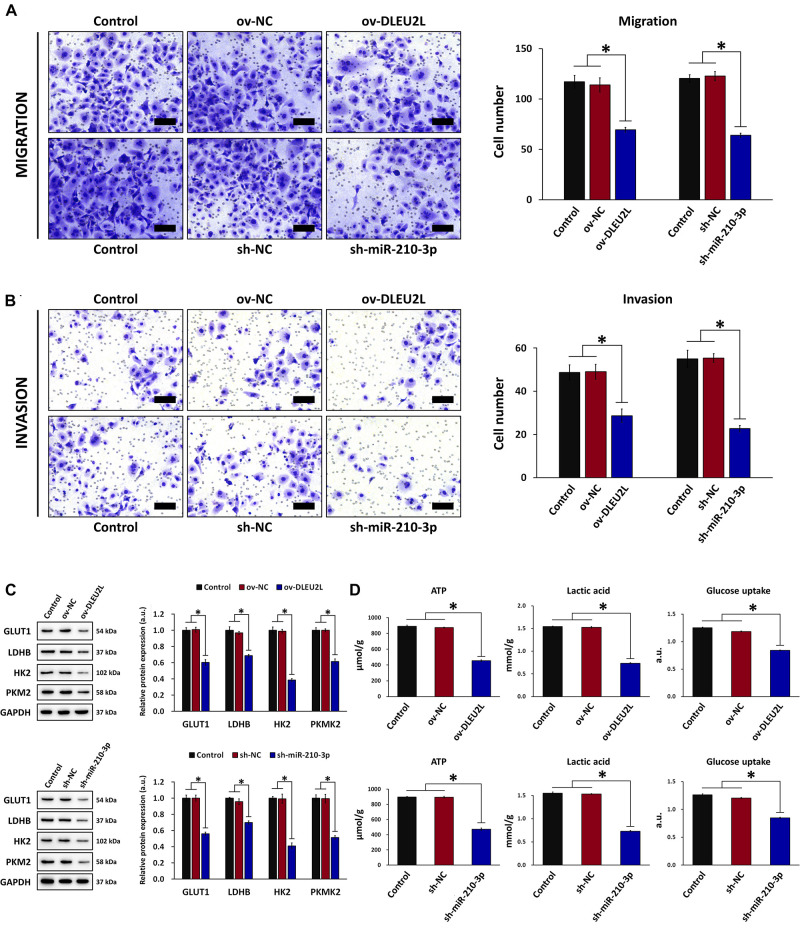
Effect of DLEU2L overexpression and miR-210-3p silencing on PANC-1 cell migration/invasion and glucose metabolism. Transwell assay and quantification of the number of **(A)** migrating and **(B)** invading PANC-1 cells, transfected with DLEU2L overexpression (ov-DLEU2L) or miR-210-3p interference (sh-miR-210-3p) vectors (or the respective negative controls), after 24 h of culture. Scale bar, 100 μm. **(C)** Western blot and quantification of the expression of proteins associated with glucose metabolism (GLUT1, LDHB, HK2, and PKM2), in PANC-1 cells transfected with DLEU2L overexpression (ov-DLEU2L) or miR-210-3p interference (sh-miR-210-3p) vectors (or the respective negative controls). **(D)** ELISA of ATP production, lactic acid production, and glucose uptake in PANC-1 cells transfected with DLEU2L overexpression (ov-DLEU2L) or miR-210-3p interference (sh-miR-210-3p) vectors (or the respective negative controls). All numerical data are expressed as the mean ± SD (*n* = 3); ^∗^*p* < 0.05. Statistical analysis was carried out using ANOVA.

Based on the results of bioinformatics analysis, we investigated whether overexpression of DLEU2L or silencing of miR-210-3p affected the biological behavior of PANC-1 pancreatic cancer cells. We first looked at the migration and invasion capability of PANC-1 cells after transfection. Upon DLEU2L overexpression (ov-DLEU2L) or miR-210-3p silencing using shRNA (sh-miR-210-3p), the number of migrating (Figure 2A) and invading (Figure 2B) cells was decreased significantly, suggesting that the transfections affected cell motility. We then examined whether glucose metabolism (the Warburg effect in tumor behavior) is influenced by transfection. The expression of glucose transporter 1 (GLUT1), lactate dehydrogenase B (LDHB), hexokinase 2 (HK2), and pyruvate kinase isozyme M2 (PKM2), was remarkably downregulated by ov-DLEU2L or sh-miR-210-3p (Figure 2C), suggesting alterations in glucose metabolism. In addition, changes in energy metabolism were assessed by evaluating ATP levels, lactic acid production, and glucose uptake. All of these were suppressed in PANC-1 cells transfected with ov-DLEU2L or sh-miR-210-3p. In all cases, the corresponding empty vectors (negative control, NC) had no effect, and the effect of ov-DLEU2L was similar to that of sh-miR-210-3p (Figure 2D).

### DLEU2L Overexpression or miR-210-3p Interference Suppressed PANC-1 Cell Proliferation and Enhanced Apoptosis via AKT/mTOR Signaling

**FIGURE 3:**
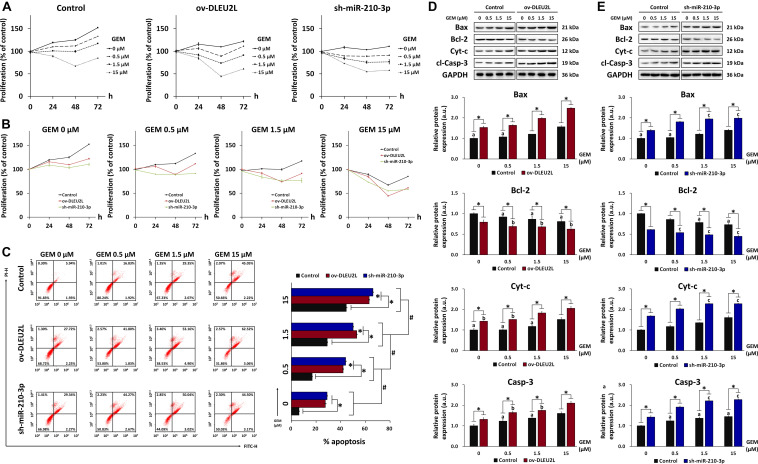
Effect of DLEU2L overexpression and miR-210-3p silencing on PANC-1 cell proliferation and apoptosis in the presence of GEM. MTT assay of PANC-1 cell proliferation plotted based on **(A)** cell transfection and **(B)** GEM concentration. For MTT assay, PANC-1 cells were non-transfected (Control) or transfected with DLEU2L overexpression (ov-DLEU2L) or miR-210-3p interference (sh-miR-210-3p) vectors and cultured in the presence of GEM at 0, 0.5, 1.5, or 15 μM for 0, 24, 48, or 72 h. **(C)** Flow cytometry of apoptosis and quantification of the percentage of late apoptotic PANC-1 cells (upper right quadrant). Western blot and quantification of the expression of proteins associated with apoptosis (Bax, Bcl-2, Cyt-c, and cl-Casp-3), in PANC-1 cells transfected with **(D)** DLEU2L overexpression (ov-DLEU2L) or **(E)** miR-210-3p interference (sh-miR-210-3p) vectors. All numerical data are expressed as the mean ± SD (*n* = 3); ^∗^*p* < 0.05; ^#^*p* < 0.05 within the same group (comparing GEM concentrations); data with the same letters in **(D)** and **(E)** are not significantly different (*p* > 0.05) in each plot of western blot. Statistical analysis was carried out using ANOVA.

We next looked at the proliferation and apoptosis of transfected PANC-1 cells in the presence of GEM at various concentrations. We first performed MTT assays for up to 72 h to determine the effect of transfection and GEM administration on the growth and survival of PANC-1 cells. As anticipated, in both non-transfected and transfected (ov-DLEU2L or sh-miR-210-3p) cells, GEM inhibited cell proliferation in a concentration-dependent manner (Figure 3A). Looking at each GEM concentration separately, we note that transfection with ov-DLEU2L or miR-210-3p both suppressed PANC-1 cell proliferation compared to non-transfection (Figure 3B). These findings are consistent with the results of Transwell invasion assay. Flow cytometry of cell cycle progression (Supplementary Figure 2) revealed that in control (non-transfected) PANC-1 cells, the proportion of cells in the G0/G1 phase increased in a GEM concentration-dependent manner. Upon DLEU2L overexpression (ov-DLEU2L) or miR-210-3p silencing using shRNA (sh-miR-210-3p), the proportion of cells at the G0/G1 phase at each GEM concentration was decreased compared to that in control cells. Concurrently, the percentage of cells in the S phase was comparatively increased, which is indicative of S phase arrest and may signify the occurrence of DNA damage via double-strand breaks (Ye et al., 2003). In addition, flow cytometry was performed to evaluate the effect of transfection on PANC-1 cell apoptosis. While the percentage of apoptosis increased significantly in a GEM concentration-dependent manner, apoptosis at each concentration was further accentuated by ov-DLEU2L or sh-miR-210-3p (Figure 3C). This finding complements the results of cell cycle progression, implicating that the overexpression of DLEU2L and silencing of miR-210-3p effectively induced PANC-1 cell death, with more prominent effects at higher GEM concentrations.

The results of flow cytometry were verified by western blot detection of apoptosis-related proteins in transfected PANC-1 cells (ov-DLEU2L or sh-miR-210-3p) treated with GEM at various concentrations (Figures 3D,E). The expression of the pro-apoptosis proteins Bax, Cyt-c, and cleaved caspase-3 (cl-Casp-3) showed a generally increasing trend with increasing concentrations of GEM. Concurrently, the expression of the anti-apoptosis protein Bcl-2 showed a concentration-dependent downward trend. Moreover, Bax, Cyt-c, and cl-Casp-3 were significantly upregulated by transfection with ov-DLEU2L or sh-miR-210-3p at all GEM concentrations, whereas Bcl-2 was significantly downregulated. In addition to revealing the concentration-dependent effect of GEM on PANC-1 cell apoptosis, the western blot observations also demonstrated the accentuating effect of DLEU2L overexpression and miR-210-3p interference on apoptosis, in the presence of GEM.

**FIGURE 4:**
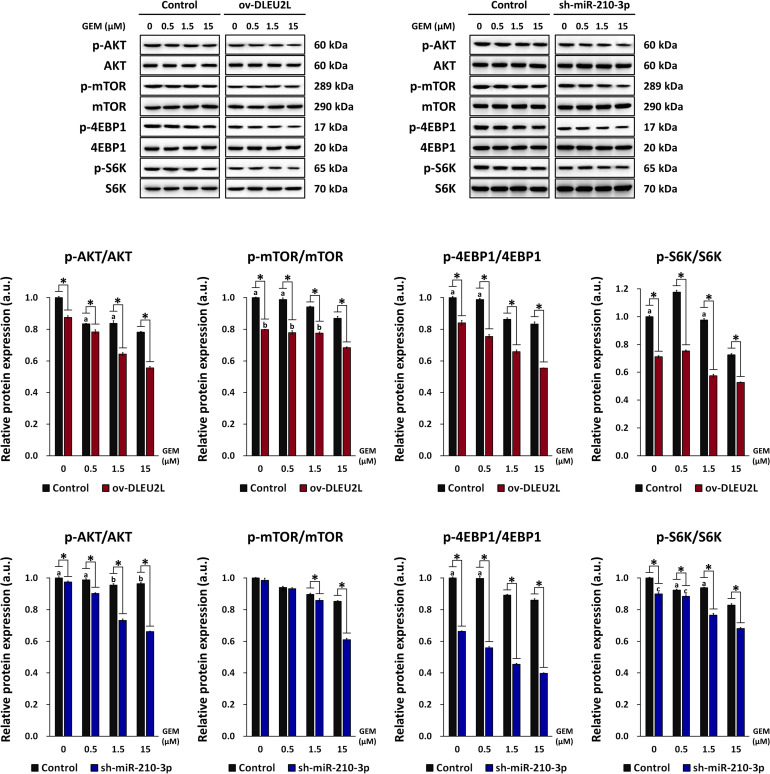
Effect of DLEU2L overexpression and miR-210-3p silencing on AKT/mTOR signaling in PANC-1 cells in the presence of GEM. Western blot and quantification of the expression of proteins associated with AKT/mTOR signaling (phosphorylation of AKT, mTOR, and S6K), in PANC-1 cells transfected with DLEU2L overexpression (ov-DLEU2L) or miR-210-3p interference (sh-miR-210-3p) vectors. Expression of phosphorylated protein was normalized to the respective total protein content. All numerical data are expressed as the mean ± SD (*n* = 3); ^∗^*p* < 0.05; data with the same letters are not significantly different (*p* > 0.05) in each plot of western blot. Statistical analysis was carried out using ANOVA.

Our previous research has revealed that exosomal miR-210 derived from GEM-resistant pancreatic cancer cells mediated the horizontal transfer of drug-resistant traits via mTOR signaling (Yang et al., 2019). We thus proposed that the tumor-suppressing properties of DLEU2L may be associated with the inactivation of the AKT/mTOR signaling pathway to impair GEM resistance. To confirm this, we examined the activation of AKT/mTOR by detecting their phosphorylation, as well as the phosphorylation of eukaryotic initiation factor 4E-binding protein (4EBP1) and ribosomal protein S6 kinase (S6K), which are downstream effectors of mTOR (Hay and Sonenberg, 2004). We observed that the levels of phosphorylated AKT, mTOR, 4EBP1, and S6K, relative to the total protein amount, showed an overall GEM concentration-dependent decrease (Figure 4). Compared to non-transfected PANC-1 cells, ov-DLEU2L or sh-miR-210-3p transfection further attenuated the levels of phosphorylated AKT, mTOR, 4EBP1, and S6K at each GEM concentration. The phosphorylation level also showed a decreasing trend in a GEM concentration-dependent manner after transfection. Considering the previously analyzed results of apoptosis, these observations signify that DLEU2L overexpression or miR-210-3p interference promoted pancreatic cancer cell death by inhibiting AKT/mTOR activation, and the effects are more prominent at higher GEM concentrations.

### DLEU2L Acts as a ceRNA by Sponging miR-210-3p to Regulate BRCA2 Expression

**FIGURE 5:**
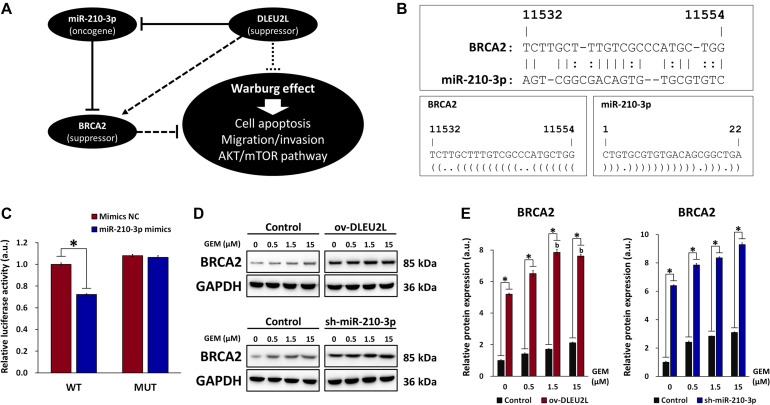
Involvement of BRCA in miR-210-3p sponging and effect on DNA damage repair. **(A)** Schematic of the proposed ceRNA sponging relationship between DLEU2L, miR-210-3p, and BRCA2 and effect on pancreatic tumor progression. **(B)** Binding sites between BRCA2 (from position 11532) and miR-210-3p, predicted by RNA22. Dot-bracket notation illustrates binding at specific nucleotides. **(C)** Dual luciferase activity assay of binding relationship between BRCA2 and miR-210-3p, using miR-210-3p mimics or negative control (NC). Relative luciferase activity represents the ratio of firefly luciferase to renilla luciferase. **(D)** Western blot of the expression of BRCA2 in PANC-1 cells transfected with DLEU2L overexpression (ov-DLEU2L) or miR-210-3p interference (sh-miR-210-3p) vectors. **(E)** Quantification of the band gray values in **(D)**. All numerical data are expressed as the mean ± SD (*n* = 3); ^∗^*p* < 0.05; data with the same letters are not significantly different (*p* > 0.05) in each plot of western blot. Statistical analysis was carried out using ANOVA.

Target genes of miR-210-3p that exhibit a possible correlation with pancreatic cancer growth and progression were screened using RNA22, as described previously. Among the target genes, BRCA2 was identified as a tumor-suppressor gene involved in DNA damage repair (Naderi and Couch, 2002). Mutations in the BRCA2 are known to be associated with increased risk of several cancer types, including pancreatic cancer (Breast Cancer Linkage, 1999; Hahn et al., 2003). Thus, proper function of BRCA2 is crucial for cancer prevention, and miR-210-3p may target BRCA2 to impair its function and promote cancer progression (Figure 5A). Binding sites between miR-210-3p and the 3′UTR of the BRCA2 gene were determined using RNA22 (Figure 5B). Based on the predicted binding sites, WT and MUT sequences of the 3′UTR of BRCA2 were designed and a dual luciferase assay was performed to verify the binding relationship between miR-210-3p and BRCA2 (Figure 5C). We observed that overexpression of miR-210-3p using mimics reduced the luciferase activity in WT-transfected cells, confirming that binding existed between miR-210-3p and the BRCA2 gene. We then confirmed that ov-DLEU2L and sh-miR-210-3p (Figures 5D,E) drastically upregulated the protein expression of BRCA2 in PANC-1 cells at all concentrations of GEM. This result is consistent with that of the dual luciferase assay. In addition, the expression of BRCA2 was increased in a GEM concentration-dependent manner in both ov-DLEU2L- and sh-miR-210-3p-transfected cells.

### DLEU2L Overexpression or miR-210-3p Interference Suppressed Xenografted Pancreatic Tumor Growth

**FIGURE 6:**
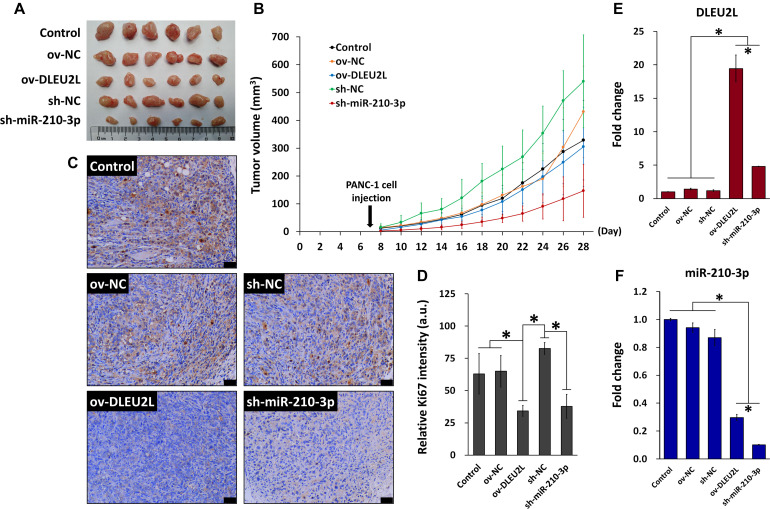
*In vivo* model of pancreatic tumor incorporating DLEU2L overexpression or miR-210-3p interference. **(A)** Xenografted pancreatic tumors extracted from mice on day 28 of tumor growth. **(B)** Tumor volume during the 28-day tumor growth period. Tumor volume was calculated using the formula volume = long diameter × (short diameter)^2^ × 0.5. **(C)** Ki-67 staining of tumor tissues. Brown staining indicates proliferating tumor cells. Scale bar, 50 μm. **(D)** Quantification of the intensity of Ki-67 staining. qRT-PCR of the expression of **(E)** DLEU2L and **(F)** miR-210-3p in tumor tissues. All numerical data are expressed as the mean ± SD (*n* = 3); ^∗^*p* < 0.05. Statistical analysis was carried out using ANOVA.

Finally, we explored the anti-tumorigenic effect of DLEU2L overexpression and miR-210-3p interference *in vivo*. A xenografted pancreatic tumor model was established in mice using PANC-1 cells, and the effect of DLEU2L overexpression or miR-210-3p interference on tumor growth was investigated. Prior to injection, PANC-1 cells were transfected with either ov-DLEU2L, sh-miR-210-3p, or their respective negative controls. After 21 days of tumor growth, we observed that tumors formed from PANC-1 cells transfected with ov-DLEU2L or sh-miR-210-3p were evidently smaller than those formed by control PANC-1 cells as well as those transfected with negative controls (Figure 6A). In particular, sh-miR-210-3p had the most prominent effect on slowing down tumor growth (Figure 6B). Ki67 staining of tumor cell proliferation revealed large areas of brown deposition in the Control, ov-NC, and sh-NC groups, which were indicative of active tumor cell proliferation. However, ov-DLEU2L and sh-miR-210-3p induced a remarkable decrease in the distribution and intensity of brown staining, implicating that tumor cell proliferation was inhibited (Figures 6C,D). We measured the expression of DLEU2L and miR-210-3p in tumor tissues (Figures 6E,F). As anticipated, ov-DLEU2L and miR-210-3p significantly upregulated the expression of DLEU2L while downregulating that of miR-210-3p compared to the three control groups. Collectively, these findings indicate that DLEU2L overexpression or miR-210-3p interference effectively disrupted pancreatic tumor growth by suppressing tumor cell proliferation.

## Discussion

The lncRNA DLEU2L is located on chromosome 1p31.3 and is also known as BCMSUN (Mertens et al., 2000). Up till now, reports on the characterization of DLEU2L remain scarce. To our knowledge, this is the first study to explore and reveal the function of DLEU2L as a ceRNA that sponges miR-210-3p to regulate BRCA2 activity and suppress pancreatic tumor progression. We previously reported the role of miR-210 as an oncogenic miRNA, the drug-resistant traits of which could be horizontally transferred from GEM-resistant pancreatic cancer stem cells to GEM-sensitive cells via exosomal delivery (Yang et al., 2019). Based on the findings of the previous study, the current investigation was designed to elucidate the upstream effector and downstream target of miR-210, further characterizing the mechanism through which miR-210 (miR-210-3p in this study) promotes pancreatic cancer resistance and progression.

Identification of a binding relationship between DLEU2L and miR-210-3p presents the interesting possibility of inhibiting miR-210-3p by overexpressing DLEU2L. Our study focused on exploring whether DLEU2L overexpression decreased the resistance of PANC-1 cells to GEM via miR-210-3p interference. This was investigated by looking at PANC-1 cell proliferation, migration, invasion, and apoptosis. Among these, the proliferative, migratory, and invasive capacities of PANC-1 cells were decreased by DLEU2L overexpression, corresponding to an increase in apoptosis. Mechanistically, we demonstrated the correlation between DLEU2L and glucose metabolism in pancreatic cancer cells. The Warburg effect is a phenomenon wherein cancer cells preferentially undergo glucose metabolism via aerobic glycolysis over oxidative phosphorylation even in the presence of abundant oxygen. The phenomenon is associated with high levels of ATP synthesis, abundant lactic acid production, and elevated glucose uptake (Goodwin et al., 2014; Liberti and Locasale, 2016). Key oncogenic factors, such as MYC and phosphatidylinositol-4,5-bisphosphate 3-kinase, have been shown to upregulate GLUTs and LDHs to promote a Warburg phenotype (Dang et al., 2009; Foster et al., 2012). In fact, the acquisition of drug resistance is closely linked to metabolic reprogramming via the Warburg effect (Grasso et al., 2017; Icard et al., 2018; Zaal and Berkers, 2018). Thus, recent research has highlighted the need to target altered glucose metabolism in cancer cells in order to improve the efficacy of chemotherapeutic drugs. Herein, the overexpression of DLEU2L in pancreatic cancer cells successfully downregulated the expression of GLUT1, LDHB, HK2, and PKM2, which are positive regulators of the Warburg effect. Meanwhile, ATP production, lactic acid content, and glucose uptake were reduced by DLEU2L overexpression. This signifies that the tumor-suppressing activity of DLEU2L is partially related to the negative regulation of metabolic reprogramming in pancreatic cancer cells.

Furthermore, we demonstrated the involvement of AKT/mTOR signaling in DLEU2L-mediated pancreatic tumor suppression. The AKT/mTOR signaling pathway has been implicated in the progression of various cancers via phenomena such as drug resistance (Deng et al., 2019), autophagy (Gao et al., 2019), and the Warburg effect (Makinoshima et al., 2015). In particular, Kagawa et al. showed that chemoresistance mediated by annexin II in GEM-resistant MiaPaCa-2 cells was associated with an upregulation of p-AKT and p-mTOR. This chemoresistant property was effectively counteracted by inhibiting mTOR phosphorylation, drawing the link between AKT/mTOR signaling and GEM resistance (Kagawa et al., 2012). Similarly, the therapeutic efficacy of GEM was enhanced via suppression of AKT/mTOR signaling when GEM was administered in combination with evodiamine (Wei et al., 2012). Our findings revealed the role of DLEU2L as an inhibitor of AKT/mTOR, as ov-DLEU2L suppressed the phosphorylation of AKT, mTOR, and the downstream effectors 4EBP1 and S6K. This inhibitory effect was mediated by miR-210-3p interference, as demonstrated in previous research (Yang et al., 2019).

Finally, we revealed that BRCA2 is a target gene of miR-210-3p and that overexpression of DLEU2L induced a drastic upregulation of BRCA2, which was presumably due to the inhibition of miR-210-3p. This validates the role of DLEU2L as a ceRNA, as it competes with BRCA2 for binding sites on miR-210-3p (Salmena et al., 2011). Overexpressed DLEU2L thus exhibits increased binding to miR-210-3p, limiting the amount of available miR-210-3p that can bind to other target genes and in turn increasing BRCA2 expression. In addition to promoting chemoresistance to GEM, miR-210 reportedly enhanced the radioresistance of human lung cancer cells by repairing DNA double-strand breaks (Grosso et al., 2013). Relevantly, BRCA2 is a crucial player in DNA damage repair pathways and is indispensable in maintaining genomic stability and integrity. In particular, BRCA2 exerts tumor-suppressing properties by mediating DNA double-strand break repair to prevent mutagenesis and eventual carcinogenesis (Liu and West, 2002; Zamborszky et al., 2017). It is important to note that GEM, as a nucleoside analog, functions by inhibiting DNA replication in cancer cells. The crosstalk between GEM and BRCA2 in terms of DNA damage is therefore a subject of particular interest. In a report by Jones et al. (2014), it was revealed that GEM-induced stalling in DNA replication is converted into double-strand breaks. Following this process, BRCA2 and RAD51, which is critically involved in homologous recombination, are recruited to assist in inhibiting replication. Unexpectedly, however, this is accompanied by the conversion of GEM-stalled forks into unrepaired double-strand breaks, further promoting GEM cytotoxicity and ultimately leading to cell death. While BRCA2 is upregulated by DLEU2L, whether the interplay between the DNA repair ability of BRCA2 and the DNA-inhibiting role of GEM is affected by DLEU2L was not investigated in this study. This will form the premise of future related research work.

## Conclusion

Knowledge on the functionality and specific properties of DLEU2L, especially its contribution to tumor progression, remains scarce. Our report provides the first evidence suggesting that DLEU2L acts as a tumor suppressor by targeting the oncogenic miR-210-3p to attenuate GEM resistance in pancreatic cancer cells. This was achieved via the AKT/mTOR signaling pathway and concurrently via the upregulation of BRCA2, which was identified as a target of miR-210-3p. Future research will focus on the in-depth DNA repair mechanisms involved in the DLEU2L-mediated regulation of GEM resistance in pancreatic cancer.

## Data Availability Statement

The raw data supporting the conclusions of this article will be made available by the authors, without undue reservation.

## Ethics Statement

The animal study was reviewed and approved by the Wuhan Myhalic Biotechnology Co., Ltd.

## Author Contributions

FX and TP: conceptualization, methodology, writing original draft, writing review, and editing. FX, HW, and JX: investigation. FX: formal analysis and visualization. TP: project administration. All authors contributed to the article and approved the submitted version.

## Conflict of Interest

The authors declare that the research was conducted in the absence of any commercial or financial relationships that could be construed as a potential conflict of interest.

## Footnotes

1 https://cm.jefferson.edu/rna22

2 http://gepia.cancer-pku.cn/index.html

3 http://starbase.sysu.edu.cn
