# Supplementary material for: Long Non-coding RNA DLEU2L Targets miR-210-3p to Suppress Gemcitabine Resistance in Pancreatic Cancer Cells via BRCA2 Regulation
Source: Front Mol Biosci. 2021 Apr 22;8:645365. doi: 10.3389/fmolb.2021.645365 (PMC8100451; doi:10.3389/fmolb.2021.645365)
Supplement: Supplementary file 1 [file Data_Sheet_1.docx]

**SUPPLEMENTARY MATERIALS**

**Long non-coding RNA DLEU2L targets miR-210-3p to suppress gemcitabine resistance in pancreatic cancer cells via BRCA2 regulation**

Fei Xu^1^, Heshui Wu^2^, Jiongxin Xiong^2^, Tao Peng^2,*^

^1^Department of Gastrointestinal Surgery, Union Hospital, Tongji Medical College, Huazhong University of Science and Technology, Wuhan, China

^2^Department of Pancreatic Surgery, Union Hospital, Tongji Medical College, Huazhong University of Science and Technology, Wuhan, China

* Corresponding author: Tao Peng

Email: pengtao8815@163.com

**Supplementary Figure Legends**

**
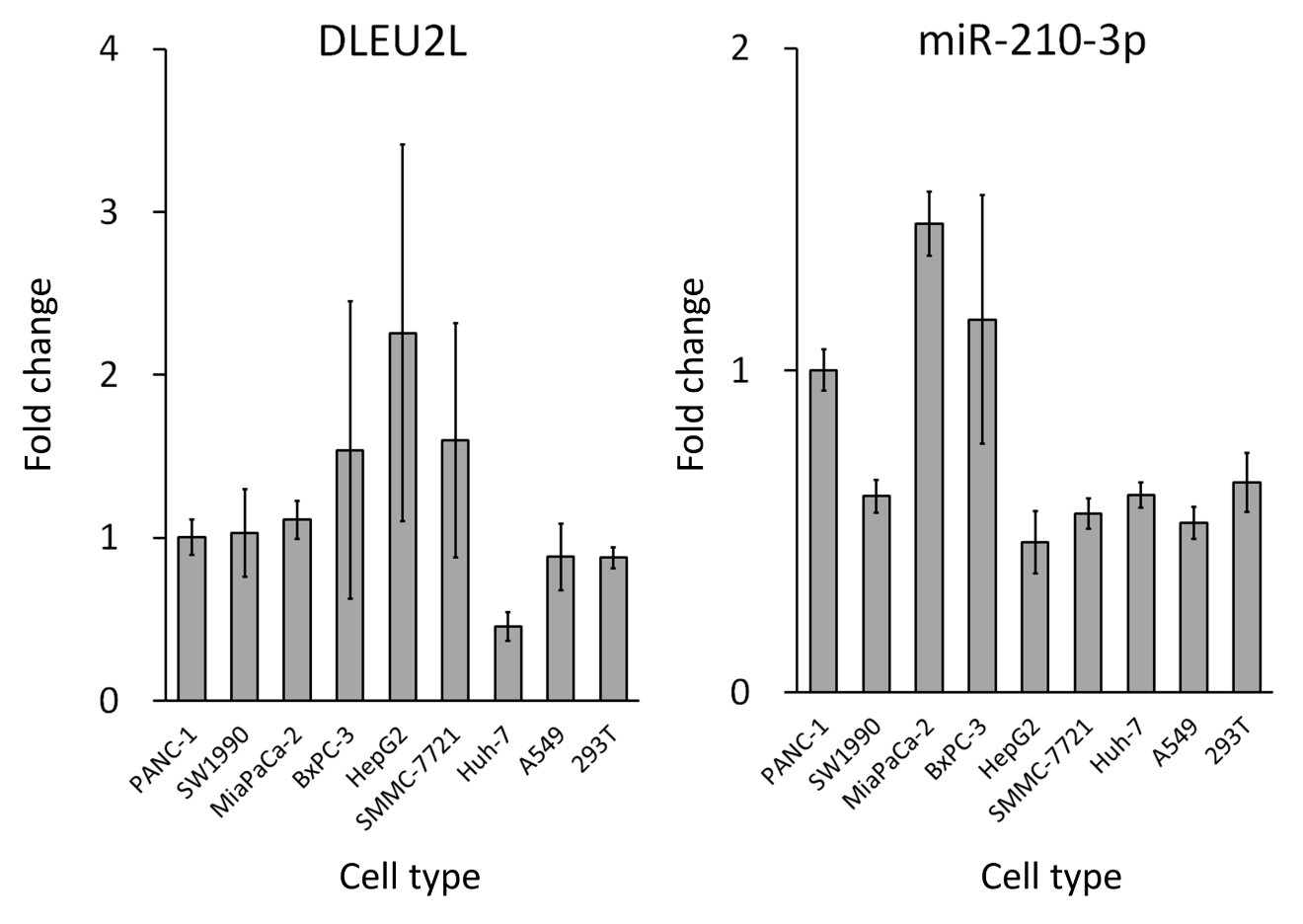
**

**Figure S1. qRT-PCR measurement of the expression of DLEU2L and miR-210-3p in different cell types.** All numerical data are expressed as the mean ± SD (n = 3).


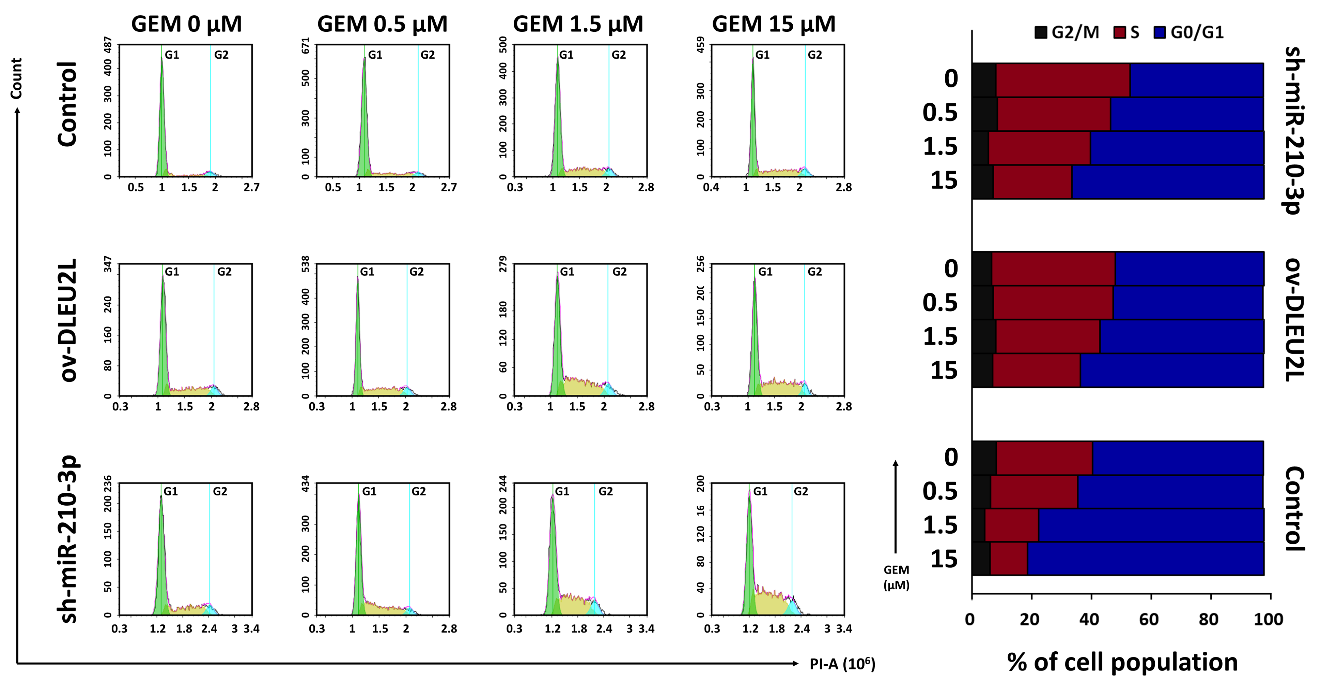


**Figure S2. Flow cytometry of cell cycle progression and quantification of the percentage of PANC-1 cells in the G2/M, S, and G0/G1 phase.** PANC-1 cells were non-transfected (Control) or transfected with DLEU2L overexpression (ov-DLEU2L) or miR-210-3p interference (sh-miR-210-3p) vectors and cultured in the presence of GEM at 0, 0.5, 1.5, or 15 μM for 0, 24, 48, or 72 h.
